# Body mass index and cognitive decline among community-living older adults: the modifying effect of physical activity

**DOI:** 10.1186/s11556-022-00284-2

**Published:** 2022-01-15

**Authors:** Isabelle Pitrou, Helen-Maria Vasiliadis, Carol Hudon

**Affiliations:** 1 Faculty of Medicine and Health Sciences, University of Sherbrooke, Charles-Le Moyne Research Center (CRCLM), 150 Place Charles-Le Moyne, Longueuil, QC J4K 0A8 Canada; 2 School of Psychology, Université Laval, CERVO Brain Research Center, 2601 chemin de la Canardière (F-2400), Québec, QC G1J 2G3 Canada

**Keywords:** Cognition, BMI, Physical activity, Older adults, Chronic disorders, Effect modification, Lifestyle

## Abstract

**Objective:**

To examine the associations between BMI categories and subsequent 3-year cognitive decline among older adults, and to test whether physical activity modifies the associations.

**Methods:**

Study sample included *n* = 1028 cognitively unimpaired older adults participating in the *Étude sur la Santé des Aînés* (ESA)-Services longitudinal study and followed 3 years later. Cognitive decline was defined as a decrease of > 3 points in MMSE scores between baseline and follow-up. BMI categories (normal weight (reference), underweight, overweight, obese) were derived from self-reported weight and height. Moderate to vigorous physical activity of ≥20 min (# of times per week) was self-reported. The presence of chronic disorders was ascertained from administrative and self-reported data. Logistic regression analyses were used to study the risk of cognitive decline associated with BMI categories stratified by weekly physical activity (≥140 min), the presence of metabolic, cardiovascular and anxio-depressive disorders.

**Results:**

In the overall sample, there was no evidence that underweight, overweight, or obesity, as compared to normal weight, was associated with cognitive decline, after adjusting for sociodemographic, lifestyle factors, and comorbidities. Individuals with overweight reporting high physical activity had lower odds of cognitive decline (OR = 0.25, 95% CI = 0.07–0.89), whereas no association was observed in individuals with overweight reporting low physical activity (OR = 0.85, 95% CI = 0.41–1.75). Among participants with metabolic and cardiovascular disorders, individuals with overweight reporting high physical activity had lower odds of cognitive decline (OR = 0.09, 95% CI = 0.01–0.59 and OR = 0.03, 95% CI = 0.01–0.92 respectively), whereas no association was observed in those with low physical activity.

**Conclusion:**

Physical activity modifies the association between overweight and cognitive decline in older adults overall, as in those with metabolic and cardiovascular disorders. Results highlight the importance of promoting and encouraging regular physical activity in older adults with overweight as prevention against cognitive decline.

**Supplementary Information:**

The online version contains supplementary material available at 10.1186/s11556-022-00284-2.

## Introduction

Population aging has led to an epidemic of chronic disorders, particularly age-related disorders as dementia. According to the 2016 Global Burden of Diseases Study, the prevalence of dementia worldwide more than doubled from 1990 to 2016 [1]. A Lancet Commission Report suggested that 12 modifiable risk factors could account for 40% of dementia related burden among which are obesity, diabetes, hypertension and physical inactivity [2]. High Body Mass Index (BMI) was cited among the top risk factors with a potential causal association with dementia [1].

Data from longitudinal population-based studies suggest that obesity in midlife is associated with an increased risk of dementia in later life [3–5]. Among older adults, study findings as to the association between BMI and cognition remain largely conflicting [5, 6]. Some have reported an obesity paradox in late-life where obesity would be protective against cognitive decline and dementia [4, 7]. A review on incident dementia, however, did not support this potential beneficial effect associated with overweight or obesity in late-life [8]. In contrast, others showed a deleterious effect of high BMI in late-life on cognition and risk of dementia [9, 10].

These previous studies highlight the complexity of the association between BMI and cognition. Among the limitations of previous reports, few examined the association between BMI and cognitive decline while taking into account important lifestyle factors [11]. Over the past years, environmental and lifestyle factors have emerged as crucial factors in the prevention or development of cognitive impairment and dementia [12, 13]. Mild to moderate alcohol use has been associated with decreased risks of cognitive impairment and dementia, whereas high levels of alcohol use and smoking were both risk factors [13]. Adopting a healthy balanced diet has been highlighted among strategies for dementia prevention [12]. Studies also highlighted the benefits of a Mediterranean diet on cognition [14]. Physical activity has been identified as an important healthy lifestyle behavior associated with better brain structure and cognition, that can contribute in reducing the incidence of dementia [2], reduce the progression of mild impairment to dementia [15] and moderate the association between adiposity and cognition [16]. In the Three City Cohort, older adults engaging in regular household and transportation activities had better executive functions and verbal fluency [17]. To finish, a recent emphasis has been placed on the beneficial effects of social connectedness, networks and social engagement on brain aging [12].

Chronic disorders as diabetes, hypertension and metabolic disorders are known predictive factors of cognitive decline also frequently associated with obesity [18–20]. Evidence however is limited regarding the impact of physical activity on cognition among older adults with chronic disorders [21, 22]. Anxio-depressive disorders have also been associated with both obesity and cognitive decline [23, 24]. If most previous studies controlled for the presence of comorbidities when examining the association between BMI and cognitive decline, mental health and psychosocial factors were often examined separately. Moreover, the literature has shown a wide variability in individual scores relating to cognitive decline [25] and reporting a statistically significant change in cognitive scores does not translate systematically into a clinically meaningful cognitive decline.

The objectives of this study were to therefore examine whether: i) BMI (normal weight (reference), underweight, overweight, obesity) at baseline was predictive of subsequent cognitive decline within a 3-year period; ii) the association between BMI and cognitive decline varies according to physical activity and the presence of chronic disorders; iii) physical activity modifies the association between BMI and cognition among older adults with metabolic, cardiovascular and anxio-depressive disorders. This study adds to the existing literature by examining BMI in late-life and 3-year cognitive decline according to physical activity level and the presence of chronic disorders.

## Methods

### Study population and data sources

This is a secondary data analysis of data drawn from the *Étude sur la Santé des Aînés-Services* (ESA-Services) longitudinal study that included a large convenience sample of *n* = 1811 Francophone older adults aged 65 years and over living in the community in the province of Quebec. The ESA-Services study participants were recruited in primary care clinics in one of the largest health regions of the province of Quebec (population of 1,507,000 inhabitants in 2016). Of the general practitioners working full-time in the region, close to 55% allowed the research team to recruit in their clinics. Patients aged ≥65 years visiting the study clinics were given a pamphlet describing the study and were invited to complete their information to be contacted for participation. Interested individuals were contacted by the study coordinator to confirm their decision and book an appointment for a face-to-face interview at their home within the month.

Structured computer-assisted interviews, averaging close to 90 min, were conducted by trained health professionals to collect sociodemographic, lifestyle and health information at baseline (T1, 2011–2013) and on average 3 years later (T2, 2014–2015). Participants gave their written informed consent to participate in the study and to access their health administrative data. Individual health survey data were linked to health administrative data from *Régie de l’Assurance Maladie du Québec* (RAMQ) and *Maintenance et Exploitation des Données pour l’Étude de la Clientèle Hospitalière* (MED-ECHO) which allowed to access physician diagnoses for medical visits and hospitalizations during the study period. The *Commission de l’Accès à l’Information du Québec* gave permission to link the datasets and the compilation was made available from data from the Government of Quebec.

The current study obtained ethics approval from the *CIUSSS Estrie – Centre Hospitalier Universitaire de Sherbrooke (*#2019–2856) ethics committee.

### Measures

#### Cognitive decline

Cognitive functioning was measured at baseline (T1) and at follow-up (T2) using a French-Canadian translation of the Mini-Mental State Examination (MMSE) adapted and validated for at home surveys in community-dwelling older adults [26, 27]. The MMSE is a 30-item short-screening instrument that assesses several cognitive dimensions being orientation, memory, calculation, language and praxis. It has shown good acceptability, feasibility and psychometric properties for the screening of cognitive impairment among community-living older adults. Possible MMSE scores range from 0 to 30 with higher scores reflecting better cognitive functioning. Cognitive decline was defined as a decline in MMSE scores of > 3 points during the 3-year study period from T1 to T2. The 3-point change in MMSE scores over a period of 3 years or more has been established as representative of a clinically meaningful decline in cognitive functioning [25] and has been defined as the minimal clinical important difference in clinical guidelines [28, 29]. To identify the onset of cognitive decline among cognitively intact individuals, participants with a MMSE score < 25 at baseline were excluded. A MMSE score of less than 25 has been validated as an accurate cut-point to distinguish normal cognitive functioning from cognitive impairment with a sensitivity of 0.87 and a specificity of 0.82 in community older adults [30]. For participants with missing MMSE scores at T2, data were imputed from administrative data. Cognitive decline was categorized as yes for *n* = 32 participants diagnosed with dementia or a dementia-related disorder within the 3 years of follow-up. The International Classification of Diseases (ICD) -9th and -10th Revision diagnostic codes used for neurocognitive disorders are summarized in Table 1.

**Table 1:** Definition of metabolic, cardiovascular, anxio-depressive and neurocognitive disorders according to administrative diagnostic codes

Chronic Disorders	Diagnostics	Administrative data	
		ICD-9 Codes	ICD-10 Codes
		(MED-ECHO)	(RAMQ)
	Hypertension	401.X; 405.X	I10.X; I15.X
Metabolic Disorders	Diabetes mellitus	250.X	E10.X; E11.X
	Dyslipidemia	272.X	E78.X
Cardiovascular Disorders	Ischaemic heart diseases and atherosclerosis	412; 413; 440.X; 441.X	I20.X; I25.2; I70.X
	Other chronic cardiac disorders	394.X; 395.X; 428.X; 427.3	I05.X; I06.X; I50.X; I48.X
Anxio-depressive Disorders	Affective disorders	296.X	F30.X; F31.X; F32.X; F33.X; F34.X; F38.X; F39
	Neurotic and stress-related disorders	300.1; 300.2. 300.4; 300.5; 300.6; 300.7; 300.8; 300.9; 311	F40.X; F41.X; F43.X; F44.X; F45.X; F48.X
Neurocognitive Disorders	Dementia and dementia-related disorders	290.X; 294.1; 331.X; 797	F00.X; F01.X; F02.X; F03; F05.1; G30.X; G31.X; G32.X; G948

Abbreviations: *ICD- 9* International Classification of Diseases -9th Revision, *ICD-10* International Classification of Diseases -10th Revision, *MED-ECHO* Maintenance et Exploitation des Données pour l’Étude de la Clientèle Hospitalière, *RAMQ* Régie de l’Assurance Maladie du Québec

#### Anthropometric data

BMI (weight in kilograms divided by height in meters squared; kg/m^2^) was computed from self-reported weight and height at baseline. Four categories were derived from the following BMI cut-offs: i) underweight for BMI < 18.5; ii) normal weight for BMI ≥ 18.5 and < 25.0, iii) overweight for BMI ≥25.0 and < 30.0, and iv) obese for BMI ≥30. These categories correspond to the Canadian guidelines for body weight classification in adults [31].

#### Lifestyle factors

Physical activity was assessed at baseline as the number of sessions of moderate to vigorous physical activity of at least 20 min per week. This single measure of physical activity has been used in previous studies among older adults [32]. High physical activity was categorized as ≥7 sessions of at least 20 min of moderate to vigorous physical activity per week, when low physical activity was defined as < 7 sessions. This categorization aimed to reflect as close as possible the Canadian Physical Activity guidelines that recommend at least 150 min of moderate to vigorous physical activity per week in adults aged 65 years and older [33].

Current dieting, smoking status, and alcohol use in the past 6 months were self-reported (yes/no questions) during the baseline interview. A score informing social support was computed based on 3 yes/no questions used in previous Canadian Community Health Surveys and referring to the presence of emotional, informational and instrumental support [34]. Social support scores range from 0 to 3 with higher scores informing higher social support.

#### Physical health

Diabetes mellitus, hypertension, dyslipidemia and cardiovascular disorders (CVD) were ascertained if present in either administrative or self-reported data. The respective ICD diagnostic codes used to ascertain diabetes mellitus, hypertension, dyslipidemia and CVD from administrative data are summarized in Table 1. Chronic physical disorders were self-reported at baseline from a list of 15 disorders as follows: arthropathy, cancer, cardiovascular, dermatologic, diabetes, eye disease, gastrointestinal, headache, hyperlipidemia, hypertension, liver disease, respiratory tract, thyroid, musculoskeletal and urinary tract disorders. The number of chronic disorders was computed as the sum of self-reported chronic physical conditions. Metabolic disorders were defined as the presence of diabetes mellitus, hypertension, or dyslipidemia. These conditions were combined as they are frequent comorbidities associated with overweight and obesity and are used in defining the presence of a metabolic syndrome [35, 36].

Participants functional status was assessed using questions from the revised Système de Mesure de l’Autonomie Fonctionnelle SMAF-IADL subscale [37, 38] which included 8 items related to the following daily tasks: cleaning the house, preparing meals, shopping, doing the laundry, using the telephone, using transportation, taking medications and managing the budget. Participants rated how they perceived their autonomy to achieve those daily tasks on 5-point Likert scales from no help needed to dependent (1 to 5). Scores were added (range 8 to 40) and categorized into low versus high functional status based on the worst quartile.

#### Mental health

Anxio-depressive disorders were ascertained if present in either administrative or self-reported data. The ICD diagnostic codes for anxio-depressive disorders are summarized in Table 1. The presence of depressive (major and minor depression) and anxiety disorders (social phobia, specific phobia, agoraphobia, panic disorder, and generalized anxiety disorder) in the past 6 months from T1 was assessed in the ESA-Services Mental Health Module based on self-reported symptoms aligned with criteria of the Diagnostic and Statistical Manual of Mental Disorders- Fifth Edition (DSM-5) [39]. The 10-item Kessler Psychological Distress scale (K10) was used to assess past-month psychological distress using 5-point Likert scales [40]. Possible scores range from 10 to 50 with higher scores indicating higher levels of distress. K10 scale has demonstrated good psychometric properties to assess distress among community-living older adults.

#### Sociodemographic factors

The following factors were considered: age (continuous variable), sex (male; female), marital status (married/marital life; single/separated/divorced/widowed), education (primary; secondary/post-secondary/university) and annual household income (< 25,000; ≥ 25,000 CAN$).

### Statistical analyses

Among the 1811 participants, 1032 were successfully interviewed at follow-up. The final analytic sample was *n* = 1028. The description of the individuals included in the analytic sample compared to those excluded or with missing data is available in Supplementary Fig. S1.

Differences in categorical and continuous variables were examined using Chi-square statistics, Fisher’s exact tests and Student’s t-tests. Logistic regression analyses were used to examine the associations between BMI categories at baseline and 3-year cognitive decline (binary outcome). BMI was categorized into 4 dummy variables as follows: (i) underweight (ii) normal weight (iii) overweight (iv) obesity, and normal weight was used as the reference category in the models. Multivariable logistic regression analyses were conducted to examine cognitive decline as a function of BMI categories in the overall sample, and successively adjusting for: a) sociodemographic characteristics (Model 1); b) sociodemographic and lifestyle factors (Model 2); c) sociodemographic, lifestyle, physical and mental health factors (Model 3). The interaction effects on cognition of BMI x physical activity, BMI x CVD, BMI x metabolic disorders and BMI x anxio-depressive disorders were also tested. Stratified analyses by physical activity and by type of chronic disorders were conducted to examine the potential differential associations of BMI categories and subsequent cognitive decline within the subgroups. Finally, to determine if the effect of physical activity was different in subgroups of individuals with chronic disorders, the analyses were stratified by physical activity level (low/high physical activity) for participants with metabolic, cardiovascular, and anxio-depressive disorders. Unadjusted and adjusted Odds Ratios (ORs) were reported with their 95% Confidence Intervals (95% CI). Statistical analyses were conducted using SAS version V 9.4 (SAS Institute, Carry, NC).

## Results

**Fig. 1:**
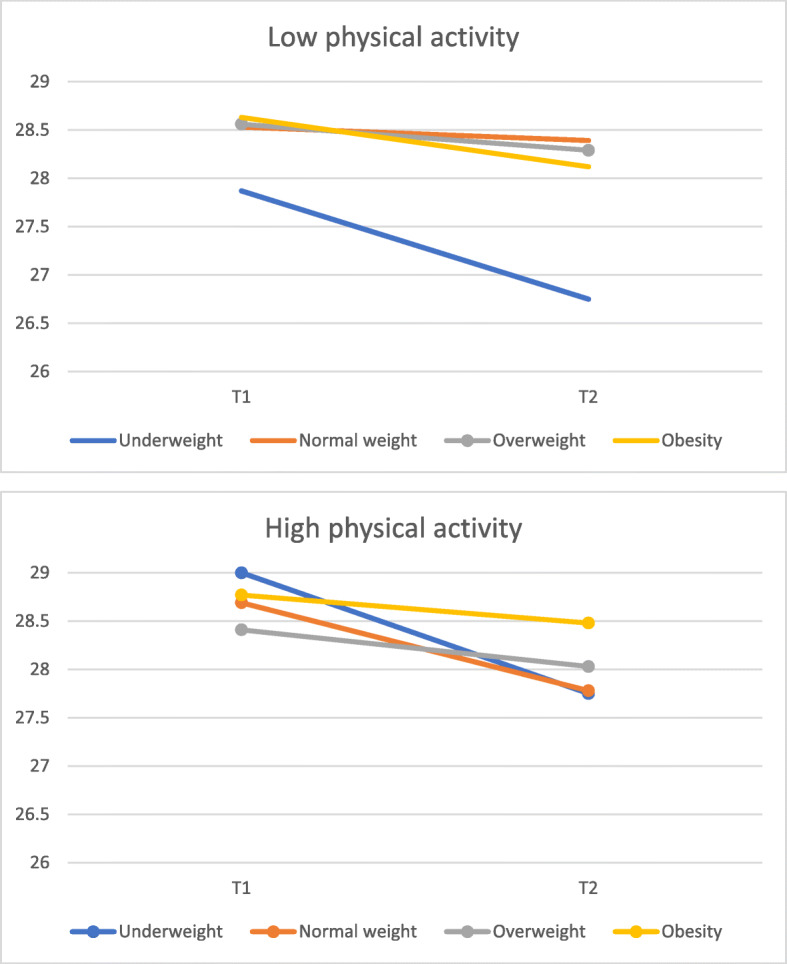
MMSE scores at T1 and T2 according to BMI categories and physical activity

During the 3-year follow-up, 85 cases of cognitive decline were identified, corresponding to an incident rate of 8.3% (95% CI = 6.6–9.9%). For participants with cognitive decline, the mean MMSE scores decreased from 28.07 ± 1.43 at T1 to 22.98 ± 2.44 at T2. Figure 1 represents mean MMSE scores at T1 and T2 by BMI categories and physical activity level. The sample characteristics according to the presence of cognitive decline are reported in Table 2. Across the BMI categories, the proportion of cognitive decline was smaller in participants with overweight and obesity (6.1 and 8.4% respectively), while the largest proportion of cognitive decline was in participants with underweight (25.0%; *p* = 0.03). There was no statistical difference between physical activity level and cognitive decline (*p* = 0.80). Supplementary Table S1 describes the sample characteristics stratified by age groups.

**Table 2:** Sample characteristics according to 3-year cognitive decline (*N* = 1028)

Variables	Cognitive decline		
	Yes (*n* = 85/1028)	No (*n* = 943/1028)	*p*-value
**BMI categories, baseline**			
Underweight	3 (25.0)	9 (75.0)	**0.03**
Normal weight	33 (10.4)	285 (86.6)	
Overweight	26 (6.1)	398 (93.9)	
Obesity	23 (8.4)	251 (91.6)	
**Sociodemographic factors**			
Age, years, mean (SD)	76.83 (5.97)	72.51 (5.77)	0.09
Sex, n (%)			
Male	33 (7.2)	424 (92.8)	0.27
Female	52 (9.1)	519 (90.9)	
Education, n (%)			
Primary	28 (13.9)	174 (86.1)	**0.01**
Secondary/Post-secondary/University	57 (6.9)	769 (93.1)	
Annual household income, n (%)			
0–25,000	28 (8.6)	298 (91.4)	0.80
≥ 25,000	57 (8.1)	645 (91.9)	
Marital status, n (%)			
Married/marital life	52 (7.8)	617 (92.2)	0.43
Single/widowed/separated/divorced	33 (9.2)	326 (90.8)	
**Lifestyle factors**			
Physical activity, n (%)			
Low: < 140 min of moderate/vigourous activity / week	64 (8.4)	698 (91.6)	0.80
High: ≥ 140 min of moderate/vigourous activity / week	21 (7.9)	245 (92.1)	
Current dieting, n (%)			
Yes	5 (5.5)	86 (94.5)	0.31
No	80 (8.5)	857 (91.5)	
Current smoking, n (%)			
Yes	7 (10.0)	63 (90.0)	0.59
No	78 (8.1)	880 (91.9)	
Alcohol use, past 6 months, n (%)			
Yes	49 (6.3)	727 (93.7)	**0.01**
No	36 (14.3)	216 (85.7)	
Social support, 1–3, mean (SD)	2.78 (0.52)	2.84 (0.48)	0.23
**Physical health**			
# Physical disorders, mean (SD)	4.27 (2.53)	3.61 (2.20)	**0.01**
Functional status, n (%)			
Low	36 (19.5)	149 (80.5)	**0.01**
High	49 (5.8)	794 (94.2)	
Diabetes, n (%)			
Yes	36 (13.5)	231 (86.5)	**0.01**
No	49 (6.4)	712 (93.6)	
Hypertension, n (%)			
Yes	61 (8.8)	630 (91.2)	0.35
No	24 (7.1)	313 (92.9)	
Hyperlipidemia, n (%)			
Yes	38 (8.5)	411 (91.5)	0.84
No	47 (8.1)	532 (91.9)	
Metabolic disorders, n (%)			
Yes	73 (8.9)	748 (91.1)	0.15
No	12 (5.8)	195 (94.2)	
Cardiovascular disorders, n (%)			
Yes	39 (10.1)	348 (89.9)	0.10
No	46 (7.2)	595 (92.8)	
**Mental health**			
Psychological distress (K10), 10–50, mean (SD)	20.77 (7.71)	17.41 (5.96)	**0.01**
Anxio-depressive disorders, n (%)			
Yes	34 (9.6)	321 (90.4)	0.27
No	51 (7.6)	622 (92.4)	

Abbreviations: # number, *BMI* Body Mass Index (in kg/m^2^)

*MMSE* Mini-Mental State Examination, *SD* Standard Deviation; **Bold:** ***p*-value < 0.05**

Table 3 reports the results of the logistic regression models for the associations between BMI categories at baseline and subsequent cognitive decline. Overall, compared to normal weight, overweight was associated with a lower risk of cognitive decline in the unadjusted model (OR _overweight_ = 0.56, 95% CI = 0.33–0.96) and in Model 1 adjusted for sociodemographic factors (OR _overweight_ = 0.56, 95% CI = 0.32–0.98). The association between overweight and cognitive decline disappeared when further adjusting for lifestyle factors (Model 2: OR _overweight_ = 0.60, 95% CI = 0.34–1.05). The associations between BMI categories and cognitive decline were not significant after full adjustment with study variables (Model 3: OR _underweight_ = 1.81, 95% CI = 0.38–8.57, OR _overweight_ = 0.63, 95% CI = 0.35–1.13 and OR _obesity_ = 0.76, 95% CI = 0.39–1.48). The interaction effects of BMI x physical activity, CVD, metabolic disorders and anxio-depressive disorders on cognitive decline were not significant in the final model.

**Table 3:** Unadjusted and adjusted Odds Ratios (ORs) of BMI categories for cognitive decline overall and stratified by physical activity and chronic disorders

	Unadjusted ORs	Model 1 ^a^	Model 2 ^b^	Model 3 ^c^
**Overall** ***(n = 85/1028)***				
Underweight	2.88 (0.74–11.16)	1.87 (0.47–7.46)	1.85 (0.44–7.85)	1.81 (0.38–8.57)
Normal weight	1.00	1.00	1.00	1.00
Overweight	**0.56 (0.33–0.96)**	**0.56 (0.32–0.98)**	0.60 (0.34–1.05)	0.63 (0.35–1.13)
Obesity	0.79 (0.45–1.38)	0.91 (0.51–1.64)	0.95 (0.52–1.73)	0.76 (0.39–1.48)
	**By Physical Activity**			
**Low physical activity** ***(n = 58/762)***				
Underweight	3.32 (0.62–17.59)	2.13 (0.38–11.91)	1.96 (0.32–11.96)	1.40 (0.20–9.64)
Normal weight	1.00	1.00	1.00	1.00
Overweight	0.71 (0.37–1.35)	0.76 (0.39–1.51)	0.82 (0.41–1.63)	0.85 (0.41–1.75)
Obesity	1.05 (0.55–2.01)	1.41 (0.70–2.84)	1.53 (0.75–3.11)	1.15 (0.53–2.50)
**High physical activity** ***(n = 21/266)***				
Underweight	2.29 (0.22–23.53)	1.38 (0.11–17.04)	2.17 (0.17–27.93)	3.19 (0.23–43.88)
Normal weight	1.00	1.00	1.00	1.00
Overweight	**0.33 (0.12–0.96)**	**0.26 (0.08–0.77)**	**0.20 (0.06–0.68)**	**0.25 (0.07–0.89)**
Obesity	0.16 (0.02–1.25)	0.18 (0.02–1.40)	0.12 (0.01–1.05)	0.11 (0.01–1.08)
	**By type of disorder**			
**Metabolic disorders** ***(n = 67/821)***				
Underweight	3.04 (0.76–12.19)	2.04 (0.50–8.36)	2.03 (0.46–8.84)	2.16 (0.44–10.54)
Normal weight	1.00	1.00	1.00	1.00
Overweight	0.58 (0.32–1.05)	0.59 (0.32–1.08)	0.59 (0.32–1.10)	0.63 (0.33–1.19)
Obesity	0.81 (0.45–1.48)	0.98 (0.53–1.84)	0.96 (0.50–1.82)	0.78 (0.39–1.59)
**No metabolic disorders** ***(n = 12/207)***				
Underweight	NA	NA	NA	NA
Normal weight	1.00	1.00	1.00	1.00
Overweight	0.48 (0.13–1.69)	0.41 (0.10–1.69)	0.61 (0.12–3.13)	0.68 (0.13–3.70)
Obesity	0.34 (0.04–2.89)	0.27 (0.03–2.58)	0.46 (0.04–4.68)	0.62 (0.05–7.34)
**Cardiovascular disorders** ***(n = 39/387)***				
Underweight	3.77 (0.64–22.35)	2.78 (0.46–16.95)	2.29 (0.33–15.75)	2.44 (0.33–18.22)
Normal weight	1.00	1.00	1.00	1.00
Overweight	0.42 (0.17–1.02)	0.41 (0.16–1.03)	0.47 (0.19–1.20)	0.48 (0.18–1.24)
Obesity	1.21 (0.56–2.65)	1.49 (0.65–3.43)	1.61 (0.68–3.82)	1.09 (0.43–2.80)
**No Cardiovascular disorders** ***(n = 46/641)***				
Underweight	1.91 (0.21–17.28)	1.31 (0.13–13.04)	1.73 (0.16–18.15)	2.45 (0.21–28.73)
Normal weight	1.00	1.00	1.00	1.00
Overweight	0.68 (0.34–1.34)	0.65 (0.32–1.33)	0.67 (0.32–1.39)	0.68 (0.31–1.45)
Obesity	0.52 (0.23–1.20)	0.54 (0.22–1.30)	0.54 (0.22–1.32)	0.46 (0.17–1.24)
**Anxio-depressive disorders** ***(n = 34/355)***				
Underweight	1.17 (0.13–10.42)	0.71 (0.08–6.65)	0.73 (0.07–7.88)	0.59 (0.05–7.47)
Normal weight	1.00	1.00	1.00	1.00
Overweight	0.63 (0.27–1.44)	0.59 (0.25–1.39)	0.71 (0.29–1.74)	0.76 (0.30–1.94)
Obesity	0.60 (0.24–1.49)	0.67 (0.26–1.75)	0.72 (0.27–1.94)	0.73 (0.26–2.06)
**No anxio-depressive disorders** ***(n = 51/673)***				
Underweight	**6.56 (1.03–41.75)**	5.35 (0.78–36.56)	5.18 (0.74–36.42)	**8.06 (1.04–62.26)**
Normal weight	1.00	1.00	1.00	1.00
Overweight	0.54 (0.27–1.08)	0.59 (0.28–1.23)	0.60 (0.29–1.26)	0.57 (0.26–1.25)
Obesity	0.94 (0.46–1.91)	1.20 (0.56–2.55)	1.25 (0.57–2.72)	0.75 (0.30–1.88)

*n/N* number of cases of cognitive decline in each strata

Abbreviations: *BMI* Body Mass Index (in kg/m^2^), *NA* Not applicable due to small cell effectives

a. Model 1 was adjusted for sociodemographic characteristics; b. Model 2 is Model 1 further adjusted for lifestyle factors; c. Model 3 is Model 2 further adjusted for physical and mental health factors; **Bold:** ***p*-value < 0.05**

A significant association between overweight and cognitive decline was observed when stratifying by physical activity. In participants reporting high physical activity, overweight was associated with a lower risk of cognitive decline in the unadjusted model (OR _overweight_ = 0.33; 95% CI = 0.12–0.96) compared to the normal weight category. The association remained statistically significant after sequentially adjusting for sociodemographic (OR _overweight_ = 0.26, 95% CI = 0.08–0.77), sociodemographic and lifestyle factors (OR _overweight_ = 0.20, 95% CI = 0.06–0.68), and sociodemographic, lifestyle and health factors (OR _overweight_ = 0.25, 95% CI = 0.07–0.89). In participants reporting low physical activity, BMI status was not associated with cognitive decline in the unadjusted and adjusted models (Model 3: OR _overweight_ = 0.85, 95% CI = 0.41–1.75 and OR _obesity_ = 1.15, 95% CI = 0.53–2.50). Among participants with no anxio-depressive disorders, underweight was associated with an increased risk of cognitive decline after adjusting for all covariables (Model 3: OR _underweight_ = 8.06, 95% CI = 1.04–62.26) compared to individuals with normal weight.

Table 4 reports the results of the multivariable regression analyses of BMI categories and cognitive decline stratified by physical activity for older adults with metabolic, cardiovascular and anxio-depressive disorders. In participants with metabolic disorders, overweight paired with high physical activity was associated with a lower risk of cognitive decline (OR _overweight_ = 0.09, 95% CI = 0.01–0.59) compared to those with normal weight and high physical activity. Among participants with metabolic disorders, overweight paired with low physical activity was not associated with cognitive decline (OR _overweight_ = 0.88, 95% CI = 0.41–1.92). Similarly, among participants with CVD, overweight paired with high physical activity was associated with a lower risk of cognitive decline (OR _overweight_ = 0.03, 95% CI = 0.01–0.92) compared to participants with normal weight and high physical activity. Among participants with CVD, overweight paired with low physical activity was not associated with cognitive decline (OR _overweight_ = 0.84, 95% CI = 0.25–2.81). Obesity was not associated with cognitive decline in those models regardless of physical activity level and type of chronic conditions.

**Table 4:** Adjusted Odds Ratios of BMI categories for cognitive decline stratified by physical activity among older adults with chronic disorders

Type of disorder	Physical activity	
	Low physical activity	High physical activity
**Metabolic disorders**	*n = 53/624*	*n = 14/197*
Underweight	1.34 (0.19–9.55)	NA
Normal weight	1.00	1.00
Overweight	0.88 (0.41–1.92)	**0.09 (0.01–0.59)**
Obesity	1.17 (0.51–2.67)	0.09 (0.01–1.15)
**Cardiovascular disorders**	*n = 26/300*	*n = 9/87*
Underweight	4.30 (0.42–43.87)	NA
Normal weight	1.00	1.00
Overweight	0.84 (0.25–2.81)	**0.03 (0.01–0.92)**
Obesity	1.83 (0.56–6.00)	0.32 (0.02–6.59)
**Anxio-depressive disorders**	*n = 27/284*	*n = 6/71*
Underweight	0.46 (0.03–6.87)	NA
Normal weight	1.00	1.00
Overweight	0.94 (0.31–2.88)	0.11 (0.01–5.40)
Obesity	0.85 (0.26–2.75)	0.74 (0.02–22.42)

Abbreviations: *BMI* Body Mass Index (in kg/m2), *NA* Not applicable due to small cell effectives

*n/N* number of cases of cognitive decline in each strata

Multivariable logistic regression models were adjusted for sociodemographic, lifestyle, physical and mental health factors; **Bold:** ***p*-value < 0.05**

## Discussion

This study examined the association between BMI and subsequent cognitive decline among older adults. In the overall sample, the association between overweight and cognitive decline disappeared after controlling for lifestyle factors, which suggests confounding and a possible modifying effect of these variables. The direction of the association between BMI in late-life and cognition remains controversial in the literature. Results from a meta-analysis indicated that overweight and obesity in late-life were associated with a respective 21 and 25% reduced risk of cognitive impairment [4]. Other studies reported that a higher BMI in late-life was associated with poorer cognitive outcomes and increased risk of dementia [9, 10]. Apart from the different samples, the divergent results in previous reports may in part be explained by the choice of potential confounding factors. Some studies controlled for sociodemographic (e.g; sex, education), psychosocial factors and mental health (e.g; anxiety and depression), while others controlled for the physical comorbidities associated with overweight and obesity (e.g; diabetes, CVD). The emphasis on lifestyle factors as possible mediators of the association between BMI and cognition is recent in contrast to obesity-related cardiovascular risk factors [41]. And if some studies controlled for smoking and alcohol use, few examined physical activity as a covariable. The current study findings highlight the importance of considering lifestyle factors, particularly physical activity, in models examining BMI and cognition.

Second, when stratifying by physical activity, overweight paired with high physical activity was associated with a lower risk of cognitive decline. The association remained statistically significant after adjusting for lifestyle and health factors. Moreover, overweight paired with low physical activity was not associated with cognitive decline. Similar patterns were observed for older adults with metabolic and cardiovascular disorders when stratifying by physical activity. The results suggest that physical activity attenuates the association between overweight and cognitive decline, particularly in older adults with chronic disorders. Our findings echo the results of Coll-Padrós et al. (2019) where overweight older adults with metabolic syndrome engaging in regular physical activity demonstrated better cognitive abilities, compared to individuals with no or low physical activity [21]. Evidence exists to support the beneficial effects of physical activity on cognition of older adults [12, 15, 42], with physical activity attenuating the relationship between central adiposity and cognition [16]. The recommendation from the American Heart Association promoting regular physical activity in older adults states that physical activity improves cognitive aging, either through a direct effect on cognition or indirectly by reducing obesity [43]. The current study also supports that physical activity is a modifier likely to have a buffering effect that mitigates the impact of overweight on cognitive decline. Among the possible physiopathological mechanisms are the known effects of physical activity on improving sensitivity to insulin, decreasing the risks of type 2 diabetes, metabolic syndrome and inflammation [42, 44].

The current findings showed an association between being underweight and increased cognitive decline among older adults without anxio-depressive disorders. Being underweight and having a decline in BMI in late-life have been associated with cognitive impairment and dementia [45]. Moreover, underweight status in older adults has been associated with higher mortality [46]. We hypothesized that in our study, individuals categorized as underweight and without anxio-depressive disorders may have worse physical health status with severe denutrition and cognitive impairment. The reverse causation where cognitive impairment leads to denutrition could also partly explain the finding.

The results we report have implications for clinical practice as for public health. These are particularly of interest given the elevated prevalence of overweight and obesity in Canada as in high-income countries [47]. The findings indicate a beneficial effect for older adults with overweight to practice regular physical activity of at least 150 min per week (equivalent to the Canadian Physical Activity guidelines) [33] as this was protective against cognitive decline. The modifying effect of physical activity on cognitive decline was also observed among participants with overweight associated with metabolic and CVD. The results highlight the benefits of adopting an active lifestyle as a prevention strategy against cognitive decline, particularly for older adults with chronic disorders. Promotion of physical activity and lifestyle interventions including exercise prescriptions [48, 49] could be implemented for older adults in primary care. Given that physical activity has numerous other beneficial health effects and can contribute to weight loss and maintenance, including regular physical activity in the clinical guidelines for older adults with overweight and obesity remains essential [50].

### Strengths and limitations

The present research has a number of strengths. First, cognitive decline was defined as a change in MMSE scores corresponding to a minimal clinically important decline [25]. Previous clinical studies used thresholds from 1.4 to 3 for MMSE score change [51]. The 3-point upper range threshold used to define cognitive decline makes it more likely that the change observed reflects a meaningful decline in cognitive abilities rather than a measurement bias. Second, the presence of chronic disorders was ascertained by using combined administrative and self-reported data. Using two data sources allowed to increase the identification of chronic physical and mental health disorders [52]. One can note that this method ascertained the presence of cases without information on the severity of disorders. Third, we examined BMI categories and their associations with cognitive decline whereas a large number of studies examined BMI continuously. This approach appears more adapted to be able to assess a U-shape rather than a linear relationship between BMI and cognition.

The findings of this study should be interpreted while considering the following limitations. First, the study included new onset of cognitive decline among cognitively intact participants. If the MMSE is a valid and reliable screening instrument to assess cognitive impairment, it is less sensitive in detecting individuals with mild impairment [53]. Some cases of mild cognitive impairments might have been consequently undetected. Second, there were some differences in the characteristics between study participants and those excluded from the analytic sample. Those excluded were older, reported a higher number of chronic conditions, a lower functional status, and higher psychological distress at baseline. In this study, we examined the association between BMI and cognitive decline among initially cognitively intact participants. These differences may reflect the characteristics of participants with cognitive impairment (older age and chronic conditions) that were excluded from the analyses rather than a selection bias. Third, data from the ESA-Services study were collected from face-to-face interviews. Self-reported measures of weight and height are subjected to biases mainly leading to underestimating BMI [54]. Among older adults, a comparison of self-reported and measured weight and height concluded of an agreement in BMI classification for about 80% of the individuals, most of the discrepancies being for individuals with cognitive impairment [55]. As participants with MMSE scores < 25 were excluded, this bias is limited. Further, to limit the potential desirability bias in reporting anthropometric data, the interviews were conducted by trained professionals in the most isolated room of the house. Third, the measure of adiposity was limited to BMI which does not discriminate between lean and fat mass. Although waist circumference and waist-hip-ratio, indicators of central adiposity [56] were not measured, metabolic disorders frequently associated with central obesity were examined according to the presence of diagnosed HTA, diabetes or dyslipidemia.

The results are generalizable to older adults with similar ethnic characteristics, as the majority were white francophones born in Canada. They may not apply to Asian populations or ethnic minorities where BMI categories are defined using other classifications [57]. Despite these limitations, the findings are of importance for future observational studies and could be easily replicated in other settings. Future studies may assess cognitive decline according to different characteristics of physical activity (types of activity, intensity, frequency, duration) and various cognitive abilities. Further research adopting a lifespan perspective are also needed to better understand the trajectories of cognitive decline. As reported in the Whitehall II study, the persistence of unhealthy behaviors from early to late midlife was associated with subsequent cognitive decline in later life [58].

## Conclusion

This research examined the associations between BMI categories and 3-year subsequent cognitive decline among community-living older adults, and the effect of physical activity on the associations. Higher level of physical activity attenuated the association between overweight and cognitive decline among older adults overall and for those with metabolic and cardiovascular disorders. The results highlight the importance of encouraging regular physical activity in older adults given its beneficial effect on cognition. Promotion of physical activity should be regarded as a preventive strategy of cognitive decline, particularly for older adults with chronic disorders.

## Supplementary Information

**Additional file 1: Fig. S1**. Study timeframe and participants’ characteristics in the analytic sample.

**Additional file 2: Table S1**. Sample characteristics according to 3-year cognitive decline stratified by age groups.

## Data Availability

The authors are not legally authorized to share or publicly publish linked survey and health administrative data due to privacy or ethical restrictions related to the use of administrative provincial health data. Requests for access to the anonymized dataset should be addressed to the ethics committee of the *CIUSSS Estrie-Centre Hospitalier Universitaire de Sherbrooke.* Participants were not requested to give informed consent for data sharing.
