# Supplementary material for: Body mass index and cognitive decline among community-living older adults: the modifying effect of physical activity
Source: Eur Rev Aging Phys Act. 2022 Jan 15;19:3. doi: 10.1186/s11556-022-00284-2 (PMC8903608; doi:10.1186/s11556-022-00284-2)
Supplement: Supplementary file 1 — Additional file 1: Fig. S1. Study timeframe and participants’ characteristics in the analytic sample. [file 11556_2022_284_MOESM1_ESM.docx]

**Supplementary fiGURE S1. Study timeframe and participants’ characteristics in the analytic sample.**

T1, 2011-2013

T2, 2014-2015

Measurements:

- Cognitive functioning (MMSE)
- BMI
- Lifestyle factors
- Chronic disorders
- Socio-demographic characteristics

Measurement:

- Cognitive functioning (MMSE)

The final analytic sample was n=1028. Compared to individuals included in the sample, those excluded or with missing data were older (73.85 vs. 72.87 years, p=0.01), reported a higher number of chronic disorders (3.92 vs. 3.67, p=0.02) and higher psychological distress at baseline (18.39 vs. 17.67, p=0.02). They were more likely to report lower education (primary level: 34.0% vs. 19.7%, p=0.01), lower household income (39.0% vs. 31.7%, p=0.01), current smoking (11.1% vs. 6.8%, p=0.01), lower functional status (34.1% vs 18.0%, p=0.01). On the other hand, they were less likely to report obesity at baseline (22.0% vs. 26.7%, p=0.02) and have a diagnosis of CVD (32.1% vs. 37.7%, p=0.02).
