# Supplementary material for: Body mass index and cognitive decline among community-living older adults: the modifying effect of physical activity
Source: Eur Rev Aging Phys Act. 2022 Jan 15;19:3. doi: 10.1186/s11556-022-00284-2 (PMC8903608; doi:10.1186/s11556-022-00284-2)
Supplement: Supplementary file 2 — Additional file 2: Table S1. Sample characteristics according to 3-year cognitive decline stratified by age groups. [file 11556_2022_284_MOESM2_ESM.docx]

**SUpplementary Table S1. Sample characteristics according to 3-year cognitive decline stratified by age groups**

| Variables | Cognitive decline,  age group: 65-74 years | | | Cognitive decline,  age group: 75-96 years | | |
| --- | --- | --- | --- | --- | --- | --- |
|  | Yes  (n=31/672) | No  (n=641/672) | p-value | Yes  (n=54/356) | No  (n=302/356) | p-value |
| **BMI categories, baseline** |  |  |  |  |  |  |
| Underweight | 1 (33.3) | 2 (66.7) | **0.01** | 2 (22.2) | 7 (77.8) | 0.72 |
| Normal weight | 14 (7.0) | 187 (93.0) |  | 19 (16.2) | 98 (83.8) |  |
| Overweight | 6 (2.2) | 263 (97.8) |  | 20 (12.9) | 135 (87.1) |  |
| Obesity | 10 (5.0) | 189 (95.0) |  | 13 (17.3) | 62 (82.7) |  |
| **Sociodemographic factors** |  |  |  |  |  |  |
| Age, years, mean (SD) | 70.4 (2.55) | 69.2 (2.83) | **0.01** | 80.5 (3.91) | 79.5 (3.98) | 0.08 |
| Sex, n (%) |  |  |  |  |  |  |
| Male | 12 (3.9) | 298 (96.1) | 0.39 | 21 (14.3) | 126 (85.7) | 0.70 |
| Female | 19 (5.3) | 343 (94.7) |  | 33 (15.8) | 176 (84.2) |  |
| Education, n (%) |  |  |  |  |  |  |
| Primary | 14 (12.5) | 98 (87.5) | **0.01** | 14 (15.6) | 76 (84.4) | 0.91 |
| Secondary/Post-secondary/University | 17 (3.0) | 543 (97.0) |  | 40 (15.0) | 226 (85.0) |  |
| Annual household income, n (%) |  |  |  |  |  |  |
| 0-25000 | 11 (5.7) | 181 (94.3) | 0.38 | 17 (12.7) | 117 (87.3) | 0.31 |
| ≥ 25000 | 20 (4.2) | 460 (95.8) |  | 37 (16.7) | 185 (83.3) |  |
| Marital status, n (%) |  |  |  |  |  |  |
| Married/marital life | 20 (4.2) | 451 (95.7) | 0.49 | 32 (16.2) | 166 (83.8) | 0.56 |
| Single/widowed/separated/divorced | 11 (5.5) | 190 (94.5) |  | 22 (13.9) | 136 (86.1) |  |
| **Lifestyle factors** |  |  |  |  |  |  |
| Physical activity, n (%) |  |  |  |  |  |  |
| Low: <140 minutes of moderate/vigourous activity / week | 19 (3.8) | 477 (96.2) | 0.10 | 45 (16.9) | 221 (83.1) | 0.11 |
| High: ≥ 140 minutes of moderate/vigourous activity / week | 12 (6.8) | 164 (93.2) |  | 9 (10.0) | 81 (90.0) |  |
| Current dieting, n (%) |  |  |  |  |  |  |
| Yes | 1 (1.5) | 65 (98.5) | 0.35 | 4 (16.0) | 21 (84.0) | 0.78 |
| No | 30 (5.0) | 576 (95.0) |  | 50 (15.1) | 281 (84.9) |  |
| Current smoking, n (%) |  |  |  |  |  |  |
| Yes | 4 (7.3) | 51 (92.7) | 0.31 | 3 (20.0) | 12 (80.0) | 0.48 |
| No | 27 (4.4) | 590 (95.6) |  | 51 (15.0) | 290 (85.0) |  |
| Alcohol use, past 6 months, n (%) |  |  |  |  |  |  |
| Yes | 16 (3.1) | 505 (96.9) | **0.01** | 33 (12.9) | 222 (87.1) | 0.06 |
| No | 15 (9.9) | 136 (96.1) |  | 21 (20.8) | 80 (79.2) |  |
| Social support, 1-3, mean (SD) | 2.7 (0.63) | 2.9 (0.45) | 0.20 | 2.80 (0.45) | 2.82 (0.54) | 0.72 |
| **Physical health** |  |  |  |  |  |  |
| # Physical disorders, mean (SD) | 4.2 (2.60) | 3.4 (2.22) | 0.10 | 4.29 (2.51) | 4.05 (2.10) | 0.50 |
| Functional status, n (%) |  |  |  |  |  |  |
| Low | 11 (12.6) | 76 (87.4) | **0.01** | 25 (25.5) | 73 (74.5) | **0.01** |
| High | 20 (3.4) | 565 (96.6) |  | 29 (11.2) | 229 (88.8) |  |
| Diabetes, n (%) |  |  |  |  |  |  |
| Yes | 12 (7.2) | 155 (92.8) | 0.07 | 24 (24.0) | 76 (76.0) | **0.01** |
| No | 19 (3.8) | 486 (96.2) |  | 30 (11.7) | 226 (88.3) |  |
| Hypertension, n (%) |  |  |  |  |  |  |
| Yes | 24 (5.6) | 401 (94.4) | 0.09 | 37 (13.9) | 229 (86.1) | 0.25 |
| No | 7 (2.8) | 240 (97.2) |  | 17 (18.9) | 73 (81.1) |  |
| Hyperlipidemia, n (%) |  |  |  |  |  |  |
| Yes | 16 (5.5) | 277 (94.5) | 0.36 | 22 (14.1) | 134 (85.9) | 0.62 |
| No | 15 (4.0) | 364 (96.0) |  | 32 (16.0) | 168 (84.0) |  |
| Metabolic disorders, n (%) |  |  |  |  |  |  |
| Yes | 28 (5.4) | 493 (94.6) | 0.12 | 45 (15.0) | 255 (85.0) | 0.84 |
| No | 3 (2.0) | 148 (98.0) |  | 9 (16.1) | 47 (83.9) |  |
| Cardiovascular disorders, n (%) |  |  |  |  |  |  |
| Yes | 14 (6.2) | 212 (93.8) | 0.16 | 25 (15.5) | 136 (84.5) | 0.86 |
| No | 17 (3.8) | 429 (96.2) |  | 29 (14.9) | 166 (85.1) |  |
| **Mental health** |  |  |  |  |  |  |
| Psychological distress (K10), 10-50, mean (SD) | 20.4 (7.58) | 17.5 (6.12) | **0.05** | 21.0 (7.87) | 17.2 (5.60) | **0.01** |
| Anxio-depressive disorders, n (%) |  |  |  |  |  |  |
| Yes | 15 (6.3) | 222 (93.7) | 0.12 | 19 (16.1) | 99 (83.9) | 0.73 |
| No | 16 (3.7) | 419 (96.3) |  | 35 (14.7) | 203 (85.3) |  |

Abbreviations: # number; BMI: Body Mass Index (in kg/m^2^); MMSE: Mini-Mental State Examination, SD: Standard Deviation; **Bold: p-value <0.05**
